# Live-Attenuated Influenza Vaccine Induces Tonsillar Follicular T Helper Cell Responses That Correlate With Antibody Induction

**DOI:** 10.1093/infdis/jiz321

**Published:** 2019-07-27

**Authors:** Sarah Lartey, Fan Zhou, Karl A Brokstad, Kristin G-I Mohn, Steffen A Slettevoll, Rishi D Pathirana, Rebecca J Cox

**Affiliations:** 1 Influenza Center, Bergen, Norway; 2 K.G. Jebsen Center for Influenza Vaccines Research, Bergen, Norway; 3 Broegelmann Research Laboratory, Department of Clinical Science, University of Bergen, Bergen, Norway; 4 Department of Research and Development, Haukeland University Hospital, Bergen, Norway

**Keywords:** antibody responses, tonsils, influenza, LAIV, T_FH_ cells

## Abstract

**Background:**

Influenza remains a major threat to public health. Live-attenuated influenza vaccines (LAIV) have been shown to be effective, particularly in children. Follicular T helper (TFH) cells provide B-cell help and are crucial for generating long-term humoral immunity. However the role of TFH cells in LAIV-induced immune responses is unknown.

**Methods:**

We collected tonsils, plasma, and saliva samples from children and adults receiving LAIV prior to tonsillectomy. We measured influenza-specific TFH-cell responses after LAIV by flow cytometry and immunohistochemistry. Systemic and local antibody responses were analysed by hemagglutination inhibition assay and enzyme-linked immunosorbent assay.

**Results:**

We report that LAIV induced early (3–7 days post-vaccination) activation of tonsillar follicles and influenza-specific TFH-cell (CXCR5+CD57+CD4+ T cell) responses in children, and to a lesser extent in adults. Serological analyses showed that LAIV elicited rapid (day 14) and long-term (up to 1 year post-vaccination) antibody responses (hemagglutination inhibition, influenza-specific IgG) in children, but not adults. There was an inverse correlation between pre-existing influenza-specific salivary IgA concentrations and tonsillar TFH-cell responses, and a positive correlation between tonsillar TFH-cell and systemic IgG induction after LAIV.

**Conclusions:**

Our data, taken together, demonstrate an important role of tonsillar TFH cells in LAIV-induced immunity in humans.

## Teaser

Live attenuated influenza vaccine induces rapid influenza-specific tonsillar TFH-cell and antibody responses in children, but is less potent in adults. The magnitude of influenza-specific TFH-cell responses inversely correlated with pre-existing salivary IgA, and directly associated with post-vaccination antibody fold-induction.

Influenza virus infects all ages and can cause major respiratory illness such as fulminant pneumonia. Influenza viruses are estimated to infect 5%–10% of adults and 20%–30% of children annually, resulting in up to 650 000 deaths globally [1]. Vaccination is the most cost-effective public health strategy to combat annual seasonal influenza [2]. Inactivated influenza vaccine (IIV) is used worldwide, and live-attenuated influenza vaccine (LAIV) is currently licensed for use in United States, Canada, and Europe (Ann Arbor backbone-LAIV), as well as in Russia and India (Leningrad backbone-LAIV). Currently used seasonal IIV induces strain-specific antibodies up to 6 months postvaccination, but it generally does not elicit broadly protective antibodies or long-lived memory B-cells [2, 3]. In contrast, LAIV elicits persistent antibodies, memory B-cell and CD4^+^ T-cell responses, as well as cross-reactive CD8^+^ T cells for up to 1 year in young children [4–8]; however, LAIV is less efficacious in adults [9]. The immunological mechanisms for the better effectiveness of LAIV in children than in adults are not fully understood.

The germinal center (GC) response is vital in the generation of high-affinity antibodies, long-lived plasma cells, and memory B cells after vaccination. Follicular T helper (T_FH_) cells are a subgroup of CD4^+^ T cells that help antigen-activated B cells through proliferation and affinity maturation inside follicles and GCs [10–17]. Follicular T helper cells express chemokine receptor CXCR5, inducible T-cell costimulator (ICOS), programmed cell death-1 (PD1), and transcriptional factor Bcl6 as canonical features. A subset of T_FH_ cells localized in follicles and GCs expresses CD57 [18, 19]. Recent studies revealed a transient T-cell type, designated as circulating T_FH_-like cells (CD4^+^CXCR5^+^CXCR3^+^ T cells [20–22] or CD4^+^CXCR5^+^PD1^+^ICOS^+^CD38^+^ T cells [23]), in the peripheral blood at 7 days after seasonal IIV. These cells provided help to memory B cells and correlated with the plasmablast and antibody responses after IIV [20–23]. However, it is not known whether LAIV elicits T_FH_-cell responses in humans, and, given their vital role in the induction of long-term humoral immunity, activation of T_FH_ cells is important in the development of new influenza vaccines.

Tonsils are located at the entrance of the upper respiratory tract and are compartmentalized organs where follicles and GCs develop in response to antigens, such as intranasally administered LAIV. We have previously shown that LAIV augments the local salivary immunoglobulin (Ig)A and tonsillar B-cell responses in children [5]. In this study, we conducted a clinical trial in children and adults to answer the following questions: (1) whether LAIV elicits T_FH_-cell responses in tonsils; (2) are there differences in the kinetics and magnitude of tonsillar T_FH_-cell responses in children and adults; (3) and whether the LAIV-induced T_FH_-cell responses correlate with local and systemic antibody responses. Here, we show that LAIV rapidly elicited T_FH_-cell and antibody responses in children and, to a lesser extent, in adults. Live-attenuated influenza vaccine-induced T_FH_-cell responses were inversely associated with pre-existing local antibodies, but they positively correlated with antibody induction after vaccination. Our findings will help to improve understanding of the immunogenicity and effectiveness of LAIV in age groups with different pre-existing immunity.

## MATERIAL AND METHODS

### Study Design

Forty children (3–17 years old) and 37 adults (18–51 years old) were enrolled in the study after recruitment from the Ear-Nose-Throat outpatient clinic at Haukeland University Hospital, Norway. All subjects were patients scheduled for elective tonsillectomy due to chronic tonsillitis, tonsillar hypertrophy, or both but otherwise healthy. The study was approved by the Ethical Committee and the Medicines Agency. All participants or their guardians provided written informed consent before inclusion in the study (NCT01866540, www.clinicaltrials.gov).

Thirty-four children and 31 adults were vaccinated with trivalent LAIV (Fluenz; AstraZeneca) during the influenza season 2013–2014. The children and adults were divided into 3 groups and vaccinated on specific days before their scheduled tonsillectomy. Age- and gender-matched unvaccinated subjects (6 children and 6 adults) were enrolled as controls.

### Vaccine and Sampling

Fluenz contained 10^7^ fluorescent focus units (FFUs) of live-attenuated A/California/7/2009-like (H1N1), A/Texas/50/2012 (Victoria-like H3N2), and B/Massachusetts/2/2012 strains. The vaccine was administered intranasally as 0.1 mL per nostril. Children under 9 years old (n = 27) received 2 doses LAIV at a 4-week interval as per the manufacturer’s recommendation. Children ≥9 years old and adults received a single LAIV dose.

Palatine tonsils were collected during tonsillectomy in phosphate-buffered saline. Tonsillar mononuclear cells (TMNCs) were isolated from 1 tonsil by Ficoll gradient centrifugation after mechanical disruption and cryopreserved at −150^o^C until use [5]. The other tonsil was cut into blocks, fixed in 4% formaldehyde, paraffin-embedded, and stored at 4^o^C until use.

Blood samples were collected before vaccination, on the day of tonsillectomy, and up to 1 year after vaccination for vaccinees, or only on the day of tonsillectomy for controls. Plasma samples were separated, aliquoted, and stored at −80^o^C until use.

Saliva samples were collected from the lower buccal mucosa before vaccination, on the day of tonsillectomy, and up to 1 year after vaccination for vaccinees using the OraSure Oral Specimen Collection Device (OraSure Technologies). The samples were stored at −80°C until use.

### Hemagglutination Inhibition Assay

Plasma was treated with receptor-destroying enzyme (RDE; Seiken) before performing preabsorption with packed turkey red blood cells (TRBCs) to remove nonspecific agglutinins. The treated plasma samples were analyzed in duplicate (starting dilution 1:10) with 8 hemagglutinating units of inactivated homologous vaccine strains and 0.7% TRBCs, as previously described [5]. The hemagglutination inhibition (HI) titer was determined as the reciprocal of the highest plasma dilution giving 50% inhibition of hemagglutination. Negative titers (<10) were assigned a value of 5 for calculation purpose.

### Enzyme-Linked Immunosorbent Assay

Influenza virus-specific Igs were quantified in plasma and saliva samples using the enzyme-linked immunosorbent assay (ELISA) developed in-house, as previously described [5]. Serially diluted plasma and saliva samples were analyzed in Maxi Sorp 96-well plates coated with 2 μg/mL split antigen from 3 vaccine viruses (kindly provided by GlaxoSmithKline, Wavre, Belgium). Immunoglobulin G, IgA, or IgM concentrations were interpolated from standard human IgG, IgA, or IgM curves, respectively. For calculation purposes, negatives were assigned as 0.05 μg/mL in plasma and 1 ng/mL in saliva.

### T-Cell Phenotyping, Stimulation, and Flow Cytometry

For T-cell phenotyping, TMNCs were thawed and rested overnight in complete Roswell Park Memorial Institute (RPMI) medium (RPMI 1640 medium containing l-glutamine, penicillin, streptomycin, and amphotericin B; Lonza) supplemented with 10% fetal bovine serum (HyClone). For influenza-specific T-cell ex vivo stimulation, rested TMNCs were incubated with 5 μg/mL split antigen from each vaccine virus and anti-CD28 (CD28.2) and anti-CD49d (9F10) antibodies, brefeldin A, and monensin (BD Biosciences).

Rested or stimulated TMNCs were stained with anti-CD3 (UCHT1), anti-CD4 (SK3), anti-CD19 (HIB19; BioLegend), anti-CD56 (NCAM16.2), anti-CD45RA (MEM-56; Thermo Fisher Scientific), anti-CXCR5 (J252D4; BioLegend), anti-CD57 (NK-1), anti-ICOS (DX29), anti-PD1 (EH12.1), and anti-CD40L (24–31; BioLegend) fluorescence-conjugated antibodies. To detect Bcl6, the surface-stained cells were fixed and permeabilized with FoxP3/Transcription factor staining buffer set (eBioscience), followed by staining with anti-Bcl6 (K112-91) fluorescence-conjugated antibody. All samples were incubated with LIVE/DEAD fixable dead cell stain kit (Thermo Fisher Scientific) and pooled human sera. All antibodies used in flow cytometry were from BD Biosciences, unless otherwise specified. Cells were acquired on LSRFortessa cell analyzer (BD Biosciences). Data were processed using FlowJo software (version 10.4.2 for Mac; TreeStar).

### Immunohistochemistry

Paraffin-embedded tonsil sections were single stained with anti-Bcl6 (PG-B6p), anti-CD4 (4B12), anti-CD20 (FL-297; Santa Cruz), anti-CD57 (TB01), or anti-ICOS (SP98; Novus Biologicals), followed by horseradish peroxidase-conjugated secondary antibody. All antibodies were from Dako, unless otherwise specified. Stained sections were counterstained with hematoxylin and analyzed using light microscopy and Cytation 5 (BioTek Instruments).

### Statistical Analyses

Biological replicates were used in all experiments, unless otherwise stated. Elevations of median fluorescence intensity from flow cytometry, HI titers, and Ig concentrations from ELISA were Ln transformed before statistical tests. Sidak’s multiple comparisons or multiple *t* tests with desired false-discovery rate of 1% were performed in a two-way analysis of variance. Non-parametric Spearman correlations were tested and linear fitting curves were plotted when Spearman *P* < .10. Fisher’s exact analysis was performed with 2 × 2 contingency table. All statistical analyses were performed with GraphPad Prism 7.

## RESULTS

### Study Design

**Figure 1.**
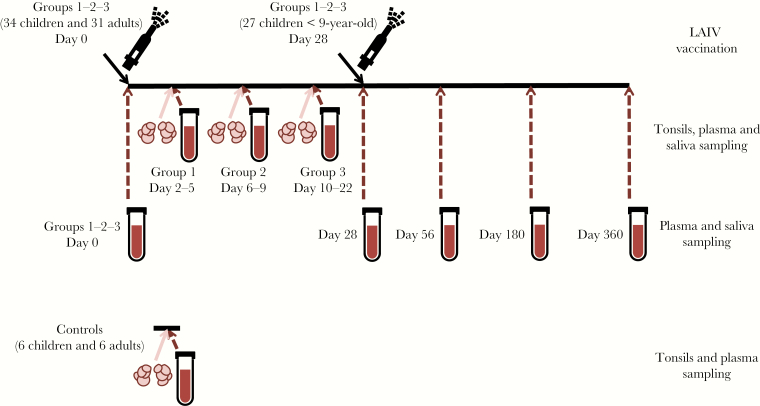
Illustration of the study design. Forty children (3 to 17 years old) and 37 adults (18 to 51 years old) were enrolled in this study. Thirty-four children and 31 adults were vaccinated with trivalent live-attenuated influenza vaccine (LAIV) (Fluenz; AstraZeneca) during the influenza season 2013–2014. The children and adults were randomized into 3 groups and vaccinated on specific days before their scheduled tonsillectomy: Group 1 (2–5 days, 7 children and 15 adults), Group 2 (6–9 days, 15 children and 8 adults), and Group 3 (10–22 days, 12 children and 8 adults). Age- and gender-matched unvaccinated subjects (6 children and 6 adults) were enrolled as controls. Children (n = 27) under 9 years old received the second dose LAIV at day 28. Tonsils, plasma, and saliva samples were collected during tonsillectomy. In addition, plasma and saliva samples prevaccination (day 0) and 28 days, 56 days, 6 months (day 180), and 12 months (day 360) postvaccination were collected for all vaccinated subjects (Groups 1–3).

Thirty-four children and 31 adults were divided into 3 groups and vaccinated with LAIV on specific days before their scheduled tonsillectomy: Group 1 (2–5 days, 7 children and 15 adults), Group 2 (6–9 days, 15 children and 8 adults), and Group 3 (10–22 days, 12 children and 8 adults) (Figure 1 and Supplementary Table). Twenty-seven children <9 years old received a second dose of LAIV 4 weeks after the first dose. Nine children and 18 adults had received the 2009 pandemic influenza vaccine, and 4 children and 5 adults had received prior seasonal influenza vaccine(s). Six unvaccinated children and 6 unvaccinated adults were enrolled as controls. None of the vaccinees or controls had received LAIV before this study. Tonsils and plasma samples were collected from all subjects on the day of tonsillectomy. Sequential pre- and postvaccination plasma and saliva samples were collected from all vaccinees (Figure 1).

### Live-Attenuated Influenza Vaccine Elicits Rapid Tonsillar Follicular T Helper Cell Responses in Children

**Figure 2.**
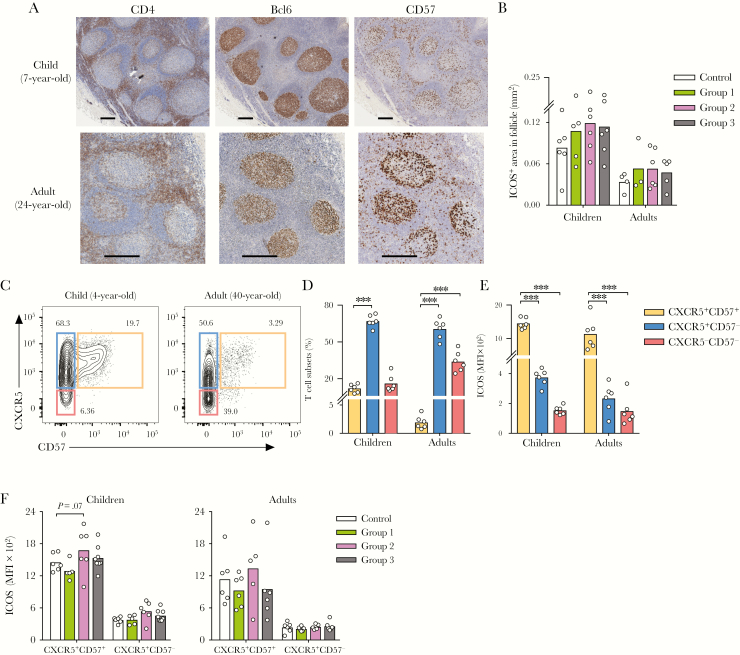
Activation of follicular T helper cells after live-attenuated influenza vaccine. (A) Representative immunohistochemistry images (original magnification ×4) of sections stained with CD4, Bcl6, or CD57 from a child (7 years old, top) and an adult (24 years old, bottom). Stained sections were counterstained with hematoxylin. Scale bar indicates 400 μm. (B) A summary of inducible T-cell costimulator (ICOS)^+^ area inside follicles from controls and vaccinees in Groups 1–3 in children (n = 22) and adults (n = 18). Follicles were measured based on CD20 and hematoxylin staining. (C) Representative CD4^+^ T-cell subsets gating based on CXCR5 and CD57 in a child (4 years old, left) and an adult (40 years old, right). Tonsillar mononuclear cells were pregated as CD3^+^CD19^−^CD56^−^CD4^+^CD45RA^−^ (pregating in Supplemental Figure S1). Numbers adjacent to outlined areas indicate frequencies of CD4^+^ T cells in each subset. (D) A summary of the distribution of the CD4^+^ T-cell subsets in 12 unvaccinated children and adults. (E) The expression of ICOS in median fluorescence intensity (MFI) in CD4^+^ T-cell subsets: CXCR5^+^CD57^+^ (orange), CXCR5^+^CD57^−^ (blue), and CXCR5^−^CD57^−^ (red) from unvaccinated children and adults. (F) A summary of ICOS expression in MFI from controls and vaccinees in Groups 1–3 in children (n = 25) and adults (n = 23). The mean values are shown as bars, and each symbol represents 1 subject (B and D–F). Sidak’s multiple comparisons between CD4^+^ T-cell subsets were performed in two-way analysis of variance ([ANOVA] D and E). Multiple *t* tests with desired false discovery rate of 1% between vaccinated and unvaccinated subjects were performed in two-way ANOVA (B and F). Data were from 6 independent experiments. ***, *P* < .001.

Immunohistochemistry (IHC) staining of tonsil sections showed that follicles (CD20^+^ area) and GCs (Bcl6^+^ area) were present in both children and adults tonsils after LAIV. CD4^+^ T cells were mostly found in T-cell zones but occasionally also within GCs. CD57^+^ cells were enriched inside GCs (Figure 2A). In this study, using IHC, we observed an increase in ICOS^+^ area inside follicles after LAIV, indicating that LAIV activated follicles in children 7–14 days postvaccination (Figure 2B). However, no change in the number or size of follicles or GCs was found (data not shown). To further study the T_FH_ cells, flow cytometry was used. Tonsillar CD4^+^ T cells were gated into 3 subsets based on CXCR5 and CD57 expression in flow cytometry. On average, 12.06% and 1.95% of CD4^+^ T cells were CXCR5^+^CD57^+^ in children and adults, respectively (Figure 2C and D). The CXCR5^+^CD57^+^ cells expressed the canonical T_FH_-cell markers ICOS, PD1, and Bcl6 (Figure 2E and Supplementary Figure S2), hence the CXCR5^+^CD57^+^ cells are bona fide T_FH_ cells [17]. Overall, children vaccinated with LAIV had higher ICOS expression on T_FH_ cells compared with controls; however, the cell frequency remained unchanged (Figure 2F and data not shown).

**Figure 3.**
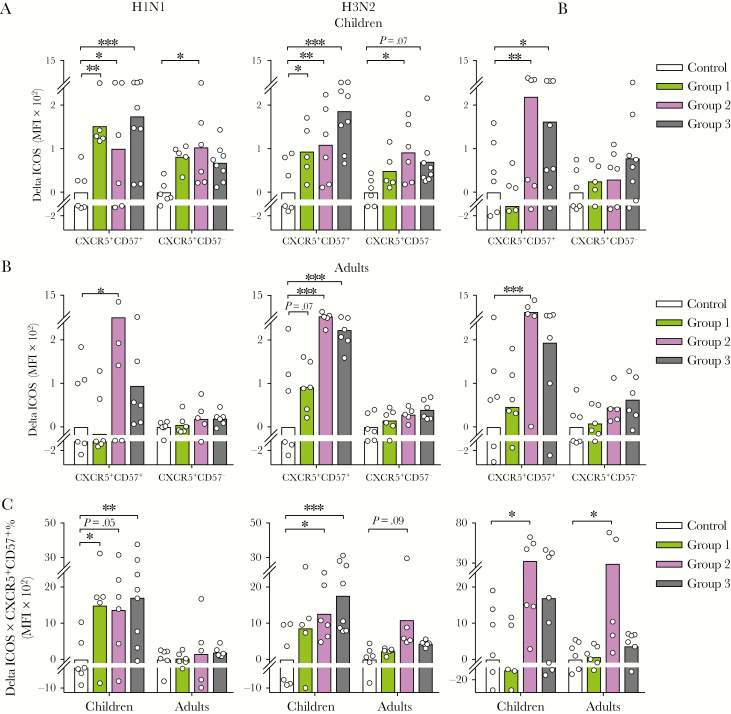
Live-attenuated influenza vaccine elicits influenza-specific follicular T helper (T_FH_)-cell responses. (A and B) Influenza-specific inducible T-cell costimulator (ICOS) expression increases (Delta ICOS in median fluorescence intensity [MFI]) in CD4^+^ T-cell subsets from controls and vaccinees in Groups 1–3 in children (n = 25; A) and adults (n = 23; B). (C) Vaccine-induced total influenza-specific T_FH_-cell responses were calculated as the elevation in T_FH_-cell activity (Delta ICOS × CXCR5^+^CD57^+^ %). Tonsillar mononuclear cells were rested and stimulated with influenza split antigens from A/California/07/2009-like (H1N1) virus (left) or A/Victoria/361/2011-like (H3N2) virus (center) or B/Massachusetts/2/2012 virus (right). Results from vaccinees (Groups 1–3) are presented as arbitrary values relative to unvaccinated subjects (Control). The mean values are shown as bars, and each symbol represents 1 subject. Multiple *t* tests with desired false discovery rate of 1% between vaccinated and unvaccinated subjects were performed in two-way analysis of variance. Data were from 6 independent experiments. *, *P* < .05; **, *P* < .01; ***, *P* < .001.

To assess influenza-specific T_FH_-cell responses after LAIV, we ex vivo-stimulated TMNCs with vaccine antigens and measured ICOS expression as a surrogate of T_FH_-cell activation [23, 24]. Children had significantly increased ICOS expression in T_FH_ cells as early as 3 days postvaccination against H1N1 and H3N2 antigens, and at day 7 against the B antigen (Figure 3A). The T_FH_ cells in adults had significantly increased ICOS expression 7 days postvaccination, against all 3 antigens (Figure 3B). Furthermore, we calculated the total LAIV-induced T_FH_-cell responses in each individual (Delta ICOS × CXCR5^+^CD57^+^ %). Live-attenuated influenza vaccine induced significant T_FH_-cell responses against all 3 viruses in children but only against B virus in adults (Figure 3C). Overall, our data showed that LAIV elicits rapid antigen-specific tonsillar T_FH_-cell responses in children and, to a lesser extent, in adults.

### Live-Attenuated Influenza Vaccine Elicits Early Antibody Responses in Children

**Figure 4.**
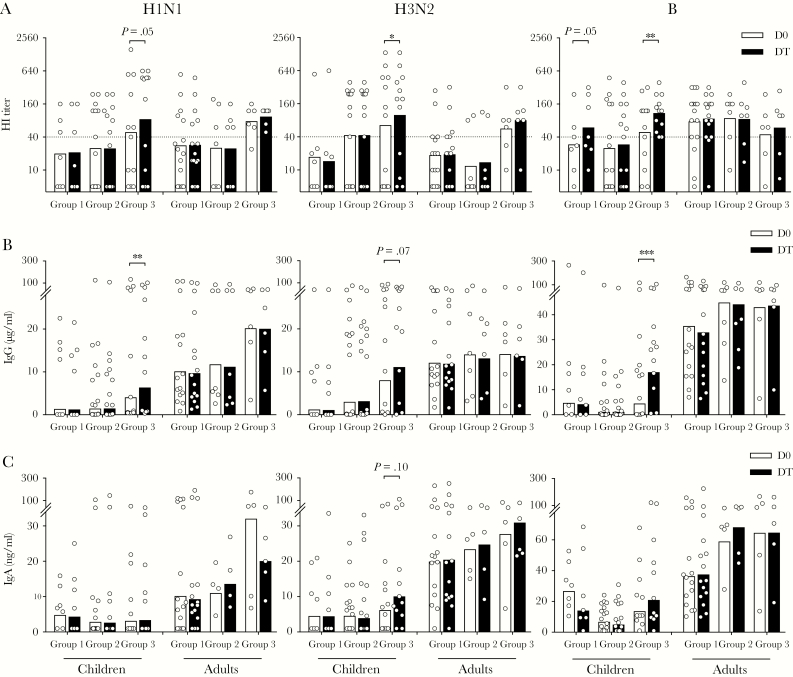
Live-attenuated influenza vaccine elicits influenza-specific antibodies within 14 days in children. Hemagglutinin-specific antibodies (hemagglutination inhibition [HI] titer; A) and total influenza-specific antibodies (immunoglobulin [Ig]G; B) were measured using plasma samples before (D0) and after vaccination (DT) from vaccinees in Groups 1–3. (C) Virus-specific local antibodies (IgA) were measured using saliva samples before (D0) and after vaccination (DT) from vaccinees in Groups 1–3. Antibodies were tested against A/California/07/2009-like (H1N1) virus (left) or A/Victoria/361/2011-like (H3N2) virus (center) or B/Massachusetts/2/2012 virus (right). The geometric mean values are shown as bars, and each symbol represents 1 subject. Antibody titers and concentrations were Ln transformed in statistical analyses. Multiple *t* tests with desired false discovery rate of 1% between pre- and postvaccination titers were performed in two-way analysis of variance. The horizontal dotted lines indicate HI titer of 40 (A). Duplicates were performed in all experiments. *, *P* < .05; **, *P* < .01.

Having established that LAIV induced rapid T_FH_-cell responses, we assessed early systemic and local antibody responses after vaccination. Hemagglutinin (HA)-specific systemic antibodies were quantified using the HI assay (Figure 4A). In children, increases in HI titers to H1N1, H3N2, and B viruses were observed as early as day 14 (Group 3) after LAIV. In adults, no increase in HI titers was observed. Neutralizing antibodies measured by microneutralization assay showed similar kinetics (data not shown). Influenza-specific antibodies were quantified using ELISA. In children, we observed increases in systemic IgG against all 3 viruses 14 days after LAIV (Figure 4B), but we only observed marginal increases in local salivary IgA against H3N2 virus (Figure 4C). Adults had higher pre-existing (D0) antibodies than children, but no increase after LAIV. Our data, taken togther, show that LAIV elicits early influenza-specific antibody responses in children, but not in adults.

### Live-Attenuated Influenza Vaccine Elicits Long-Term Antibody Responses in Children

**Figure 5.**
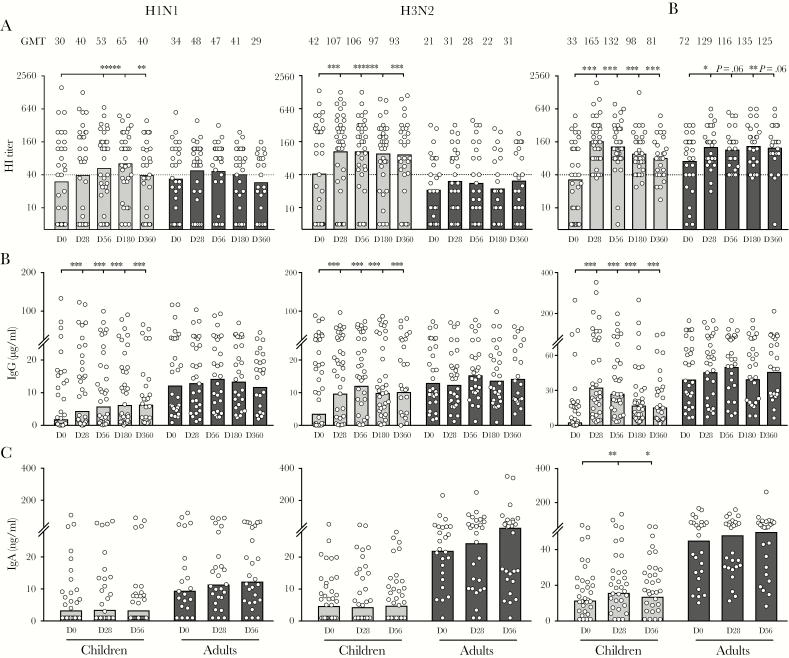
Live-attenuated influenza vaccine elicits long-term influenza-specific systemic and local antibodies in children. Systemic hemagglutinin-specific antibodies (hemagglutination inhibition [HI] titer; A) and total influenza-specific antibodies (immunoglobulin [Ig]G; B) were measured using plasma samples prevaccination (D0) and 28 days (D28), 56 days (D56), 6 months (D180), and 12 months (D360) postvaccination. Local influenza-specific antibodies (IgA; C) were measured using saliva samples before vaccination (D0) and 28 days (D28) and 56 days (D56) after vaccination. Antibodies were tested against A/California/07/2009-like (H1N1) virus (left) or A/Victoria/361/2011-like (H3N2) virus (center) or B/Massachusetts/2/2012 virus (right). The geometric mean titers are shown as bars, and each symbol represents 1 subject. Antibody titers and concentrations were Ln transformed in statistical analyses. Sidak’s multiple comparisons between prevaccination (D0) and each time point postvaccination (D28, D56, D180, and D360) were performed in two-way ANOVA. The geometric mean HI titers (GMT) at each time point are noted above the graphs, and the horizontal dotted lines indicate HI titer of 40 (A). Duplicates were performed in all experiments. *, *P* < .05; **, *P* < .01; ***, *P* < .001.

We assessed the antibody responses up to 1 year after LAIV. After immunization, 6, 10, and 19 of 34 children, and 3, 2, and 5 of 31 adults seroconverted (≥4-fold increase from prevaccination HI titers) to H1N1, H3N2, and B viruses, respectively. The HI titers after LAIV were maintained above protective titer (≥40) for 1 year in children against all 3 viruses but only against influenza B in adults (Figure 5A).

We quantified the influenza-specific systemic IgG (Figure 5B), IgA, and IgM (Supplemental Figure S3). In children, LAIV elicited significant increases in IgG against all 3 antigens at 28 days postvaccination, which were maintained throughout the year. In contrast, adults showed no increase in antibodies after LAIV (Figure 5B). Similar trends, although of lower magnitudes, were observed with IgA responses (Supplemental Figure S3A). Live-attenuated influenza vaccines also induced an IgM response in children against influenza B (Supplemental Figure S3B).

Next, we measured local salivary antibodies up to 56 days after vaccination. Live-attenuated influenza vaccine significantly increased the local IgA to influenza B virus in children; however, no change was observed in adults (Figure 5C). In summary, our data showed that in children, LAIV induced long-term antibody responses to all 3 viruses, but local antibodies were only elicited against influenza B virus. In adults, only influenza B-specific antibody responses were observed after LAIV.

### Follicular T Helper Cell Responses After Live-Attenuated Influenza Vaccine Correlated With Antibody Responses

**Figure 6.**
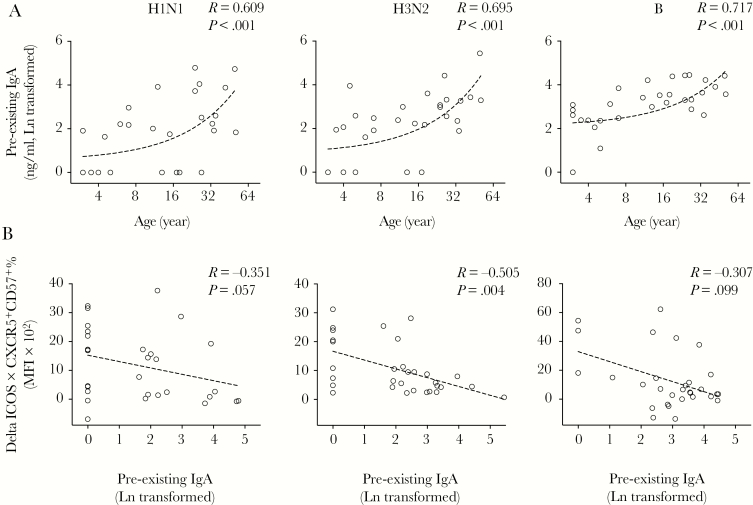
Pre-existing local antibodies correlate with age and follicular T helper (T_FH_)-cell responses after live-attenuated influenza vaccine (LAIV). (A) Pre-existing influenza-specific local antibodies (immunoglobulin [Ig]A) in saliva samples from children and adults correlated strongly with their ages. (B) The LAIV-induced T_FH_-cell responses (Delta inducible T-cell costimulator [ICOS] × CXCR5^+^CD57^+^ %) inversely correlate with pre-existing influenza-specific local antibodies (IgA). The T_FH_-cells activity and antibodies were tested against split antigens from A/California/07/2009-like (H1N1) virus (left) or A/Victoria/361/2011-like (H3N2) virus (center) or B/Massachusetts/2/2012 virus (right). Pre-existing IgA concentrations in saliva samples were Ln transformed in statistical analyses. Linear fitting curve was plotted as dotted line when nonparametric Spearman *P* < .10. Spearman r and *P* values are noted for each correlation. MFI, median fluorescence intensity.

Our next aim was to assess whether LAIV-induced T_FH_-cell responses correlated with the age of the subject or pre-existing local antibody levels. First, we observed an increase in the pre-existing influenza-specific salivary IgA concentration with an increase in age (Figure 6A). Notably, pre-existing salivary IgA concentrations inversely correlated with LAIV-induced T_FH_-cell responses (Figure 6B), suggesting that high influenza-specific local IgA concentrations present in adults may limit the tonsillar T_FH_-cell responses.

**Figure 7.**
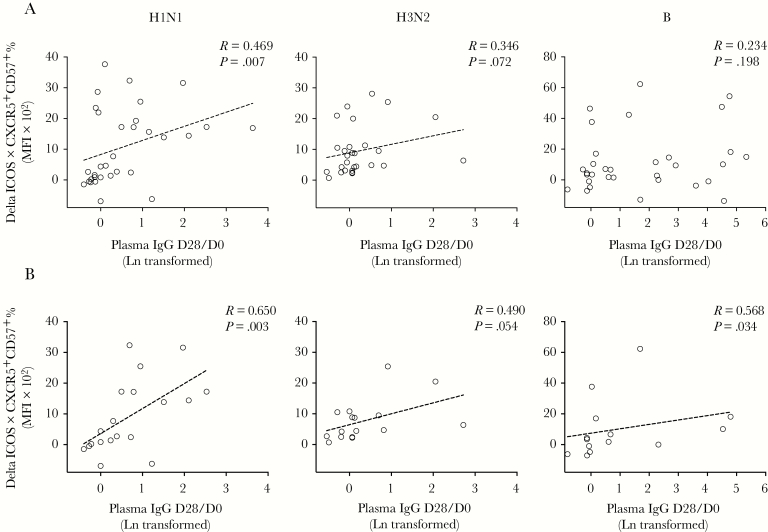
Influenza-specific follicular T helper (T_FH_)-cell responses after live-attenuated influenza vaccine (LAIV) correlate with antibody responses. (A) The correlations between LAIV-induced T_FH_-cell responses (Delta inducible T-cell costimulator [ICOS] × CXCR5^+^CD57^+^ %) and systemic antibody fold induction (plasma immunoglobulin [Ig]G D28/D0) from all vaccinated children and adults. (B) Influenza A viruses specific T_FH_-cell and antibody responses correlate in naive individuals (D0 hemagglutination inhibition [HI] <40), whereas influenza B virus-specific T_FH_-cell and antibody responses correlate in pre-exposed individuals (D0 HI ≥40). The T_FH_-cell responses and antibody fold induction were tested against split antigens from A/California/07/2009-like (H1N1) virus (left) or A/Victoria/361/2011-like (H3N2) virus (center) or B/Massachusetts/2/2012 virus (right). Systemic antibody fold inductions (plasma IgG D28/D0) were Ln transformed in statistical analyses. Linear fitting curve was plotted as dotted line when nonparametric Spearman *P* < .10. Spearman r and *P* values are noted in each correlation. MFI, median fluorescence intensity.

Next, we tested whether tonsillar T_FH_-cell responses elicited after LAIV correlated with systemic antibody induction postvaccination. It is interesting to note that LAIV induced influenza-specific T_FH_-cell responses correlated with the systemic IgG fold-induction 28 days after vaccination (D28/D0), but not HI titers, against H1N1 and H3N2 antigens (Figure 7A). We further dissected antibody responses by stratifying vaccinees as naive (HI titer <40) or experienced (HI titer ≥40) before vaccination. We were intrigued to find that T_FH_-cell and antibody responses correlated more closely in naive individuals for influenza A (H1N1 and H3N2) viruses. In contrast for influenza B virus, T_FH_-cell and antibody responses significantly correlated only in experienced vaccinees (Figure 7B). Similar patterns were also observed with antibody responses 56 days after LAIV (Supplemental Figure S4). The correlations between T_FH_-cell and systemic IgG responses were confirmed using Fisher’s exact test (Supplemental Figure S5). Our data, taken together, demonstrate that LAIV-induced tonsillar T_FH_-cell responses correlated with systemic antibody responses after vaccination.

## DISCUSSION

The mechanisms by which vaccines, particularly live-attenuated viral vaccines, elicit persistent antibody responses remains unclear. Understanding the underlying immunological mechanisms that mediate long-term immunity is critical in designing improved vaccines. Follicular T helper cells play a critical role in GC formation and the development of high affinity antibody and B-cell responses [13], thus vaccines that successfully promote and maintain T_FH_ cells are most likely to induce persistent antibody responses. Our data show, for the first time, that LAIV rapidly activated follicles and tonsillar T_FH_ cells in children and, to a lesser extent, in adults. We also observed rapid and long-term (up to 1 year) antibody responses to all 3 vaccine viruses in children, but only to influenza B virus in adults. The magnitude of tonsillar T_FH_-cell responses was inversely correlated with pre-existing local IgA antibodies. More important, significant correlations were observed between systemic antibodies and tonsillar T_FH_-cell responses after LAIV.

Recent studies have shed light on the tonsillar T_FH_-cell responses against influenza in humans [25, 26]. In children receiving IIV, Amodio et al [25] found a significant increase in the frequencies of T_FH_ cells, which were associated with influenza-specific, antibody-secreting cells. Aljurayyan et al [26] in vitro stimulated TMNCs from unvaccinated individuals with attenuated influenza viruses and reported T_FH_-cell activation, proliferation, and differentiation, which correlated with antibody production. In our study, using IHC, we showed that a substantial number of CD4^+^ and CD57^+^ cells are present inside follicles, in agreement with previous observations [18, 19]. Immunohistochemistry showed that follicles inside tonsils were activated after LAIV, indicated by the elevated ICOS expression. However, no change in follicle number or size was found after vaccination, suggesting the absence of a strong GC reaction. Flow cytometric analyses also showed higher ICOS expression in the T_FH_-cell (CXCR5^+^CD57^+^CD4^+^ T cell) population, suggesting increased T_FH_-cell activation, although the T_FH_-cell frequencies remained unchanged after LAIV. The lack of increase in T_FH_-cell frequencies after LAIV vaccination may be attributed to the replicating nature of the LAIV with a relatively low antigen dosage (10^7.0 ± 0.5^ FFU) compared with IIV (commonly 15 μg of HA per strain). However, when LAIV-specific T_FH_-cell responses were ex vivo stimulated, we observed potent T_FH_-cell responses against all 3 LAIV viruses in children and against the B virus in adults (Figure 3). Future studies on tonsillar T_FH_-cell and circulating T_FH_-like-cell responses after LAIV will help gain insights into the relationship between these lymph node and systemic T_FH_ cells and to establish whether circulating T_FH_-like cells are a possible biomarker for the immunogenicity of LAIV.

To dissect humoral responses after LAIV, we quantified systemic influenza-specific antibodies as HA-specific functional and binding antibodies in plasma samples. In addition, local IgA were measured in saliva samples (Figures 4 and 5). The HI titer of 40 has been widely used as a surrogate correlate of protection [27], although an HI titer of 110 was suggested for protection against H3N2 in children [28]. Recent studies have shed light on the protective roles of nonneutralizing antibodies. For example, neuraminidase and internal protein-specific antibodies may contribute to elimination of infection through limiting progeny virion release and mechanisms such as antibody-dependent, cell-mediated cytotoxicity [29, 30]. Meanwhile, mucosal IgA provides protection against the initial establishment of infection [4]. We found significant increases in influenza-specific antibodies 14 days after LAIV, which were maintained for up to 1 year in children to all 3 viruses. However, in adults, LAIV only elicited antibodies to the B virus. These differences in the immunogenicity among vaccine viruses observed are consistent with previous findings [31], and they could be due to differences in virus replication efficiency in the upper respiratory tract. In addition, although we only observed low local IgA responses after LAIV using saliva samples (Figures 4 and 5), future studies could measure IgA in nasal washes to gain more direct evidence of mucosal antibody responses after LAIV.

During the 2009 pandemic, Norway vaccinated its population with AS03-adjuvanted pandemic vaccine, which induced durable antibody responses [32]. Among the vaccinees in our cohort, 27% of children and 52% of adults had received this pandemic vaccine. We observed higher pre-existing H1N1 HI titers in these individuals (*P* = .03 in children and *P* = .002 in adults) (Supplemental Figure S6), consistent with our previous findings [33]. The higher frequency of adults receiving pandemic vaccine may help explain the higher H1N1-specific pre-existing antibodies and subsequently the lower antibody responses after LAIV in adults compared with children. In future studies, a comparison of T_FH_-cell responses elicited by IIV and LAIV may provide better insight into understanding differences in the immune responses elicited by parenteral and intranasal influenza vaccines.

In Europe, LAIV is only licensed and recommended for children 2–17 years old, because LAIV elicits more potent and longer-lasting immune responses in children than in adults [2, 33]. Our analyses revealed 3 correlations: (1) the pre-existing local IgA correlates with age; (2) LAIV-induced T_FH_-cell responses inversely correlate with the pre-existing local IgA; and (3) systemic antibody responses after vaccination correlate with T_FH_-cell responses. Our data, taken together, may explain why LAIV works better as priming vaccine in children [34]. The low pre-existing local antibody levels at the site of LAIV administration in children may aid virus replication, thus leading to the generation of rapid T_FH_-cell responses and subsequent long-term humoral responses after vaccination. In contrast, higher pre-existing local antibodies in older individuals, likely due to previous exposure or vaccination, may partially inhibit the initial LAIV virus replication. As a result, LAIV elicits lower T_FH_-cell activation in adults, which provides insufficient B-cell help, and consequently lower humoral responses after vaccination. We found that LAIV-induced T_FH_-cell responses inversely correlated more strongly with pre-existing local IgA than age, implying that LAIV functions optimally as a priming vaccine on no or low background immunity. It is interesting to note that T_FH_-cell and antibody responses correlate significantly in naive vaccinees with H1N1 and H3N2 viruses but with B virus in pre-exposed vaccinees. This could be explained by influenza B virus being better adapted to humans than influenza A viruses, thus lower T_FH_-cell activation is needed for antibody induction (as confirmed in the Fisher’s exact test) (Supplementary Figure S5). Further studies with a larger cohort are needed to validate our findings.

## CONCLUSIONS

In this study, we assessed the kinetics and magnitude of LAIV-induced T_FH_-cell, systemic, and local antibody responses in children and adults. In this study, for the first time, we have shown that LAIV elicits rapid tonsillar T_FH_-cell responses to all 3 influenza vaccine viruses, which correlated with the long-term systemic antibody increases after vaccine. Our data will contribute to the understanding of mechanisms that govern LAIV-induced immune responses in pediatric and adult populations.

## Supplementary Data

Supplementary materials are available at *The Journal of Infectious Diseases* online. Consisting of data provided by the authors to benefit the reader, the posted materials are not copyedited and are the sole responsibility of the authors, so questions or comments should be addressed to the corresponding author.

jiz321_suppl_Supplementary_Figure

jiz321_suppl_Supplementary_Figure_Legend

jiz321_suppl_Supplementary_Table

## Notes

***Acknowledgments.*** We thank the patients (and their parents) involved in this study, Dr. Camilla Tøndel and the staff at the Pediatric Clinical Trials Unit, and Dr. Hans Jorgen Aarstad and staff at the Ear, Nose and Throat Department, Haukeland University Hospital, Norway and the staff at the Influenza Centre, Department of Clinical Sciences, University of Bergen, Norway.

***Author contributions.*** F. Z., R. D. P., and R. J. C. conceived the project and designed the experiments. R. J. C., K. G.-I. M., and K. A. B. conducted the clinical study and collected samples. F. Z., S. A. S., and S. L. conducted the experiments. F. Z. and S. L. analyzed the data. F. Z., S. L., K. A. B., K. G.-I. M., R. D. P., and R. J. C. prepared and edited the manuscript.

***Financial support.*** The study was funded intramurally by the Influenza Centre at the University of Bergen and Haukeland University Hospital. The Influenza Centre is funded by the Ministry of Health and Care Services, Norway, the Norwegian Research Council Globvac (284930), the European Union (Univax 601738 and EU IMI115672, FLUCOP), EU Nanomedicines Flunanoair (ERA-NETet EuroNanoMed2 i JTC2016), Helse Vest, and the K.G. Jebsen Centre for Influenza Vaccine Research.

***Potential conflicts of interest.*** All authors: No reported conflicts of interest. All authors have submitted the ICMJE Form for Disclosure of Potential Conflicts of Interest.
