# Supplementary figures and images for: Live-Attenuated Influenza Vaccine Induces Tonsillar Follicular T Helper Cell Responses That Correlate With Antibody Induction
Source: J Infect Dis. 2019 Jul 27;221(1):21–32. doi: 10.1093/infdis/jiz321 (PMC6910880; doi:10.1093/infdis/jiz321)

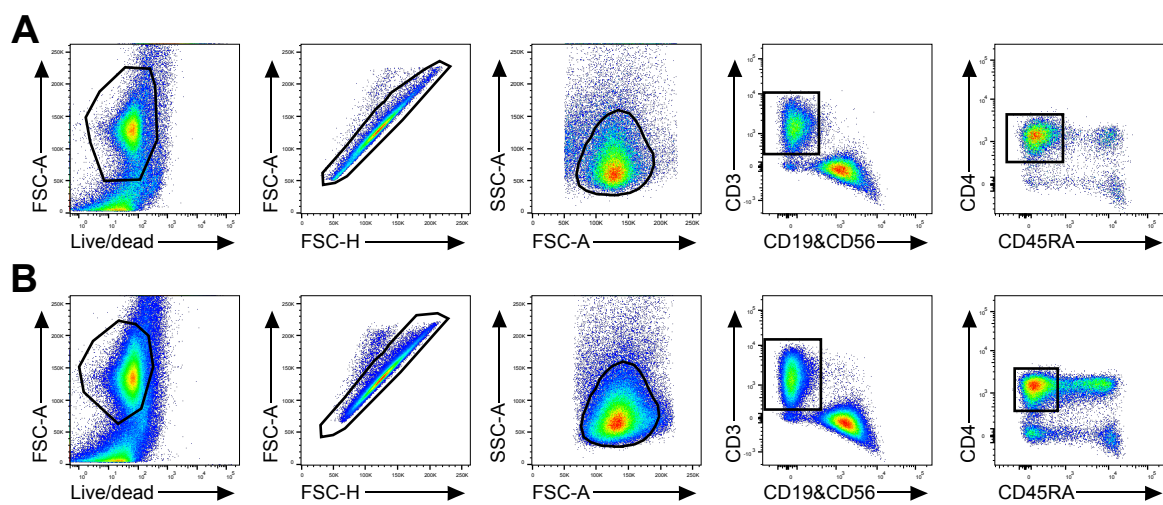

Figure S1

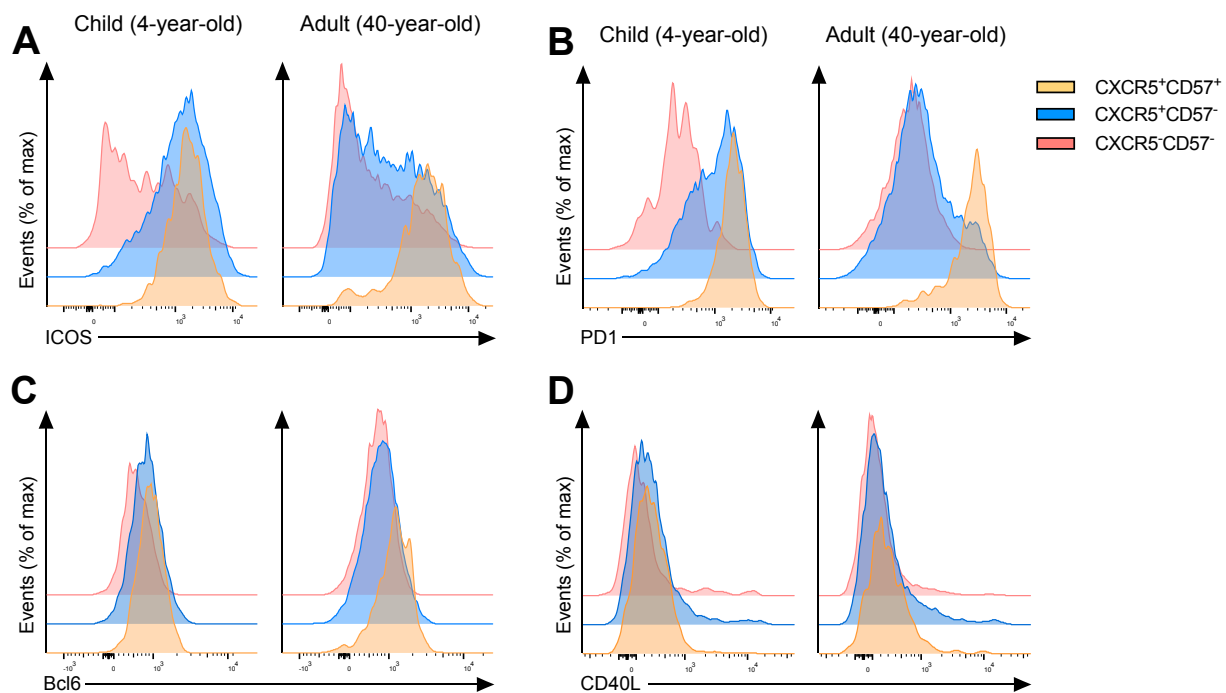

Figure S2

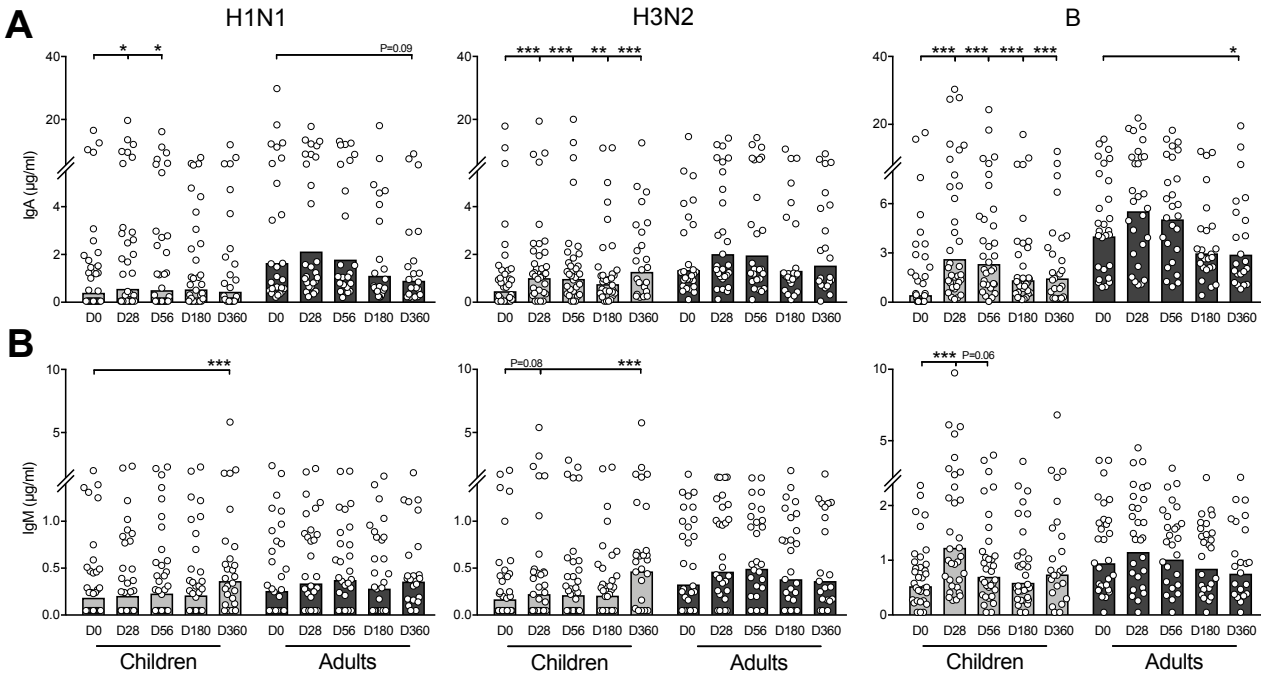

Figure S3

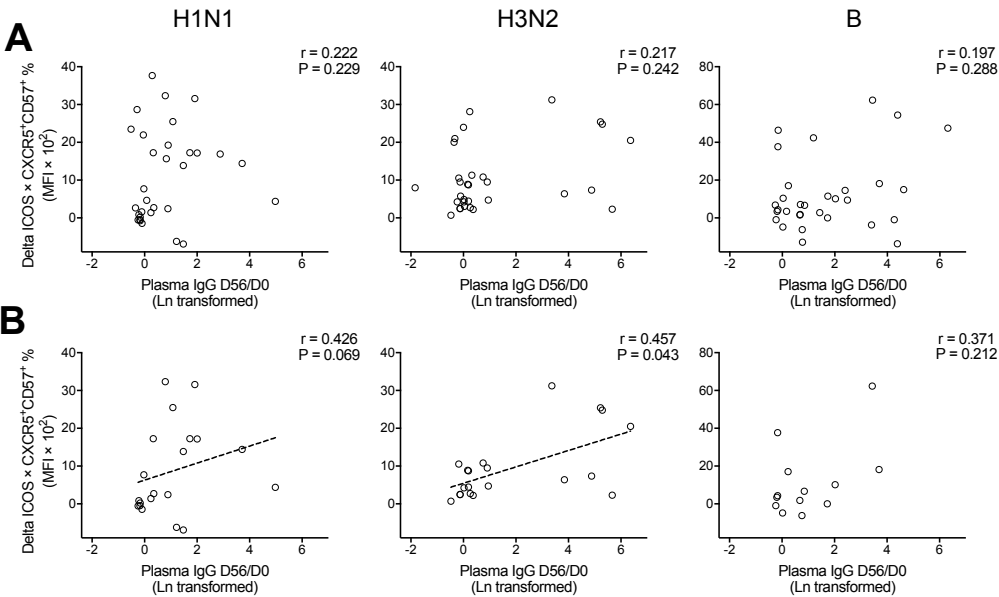

Figure S4

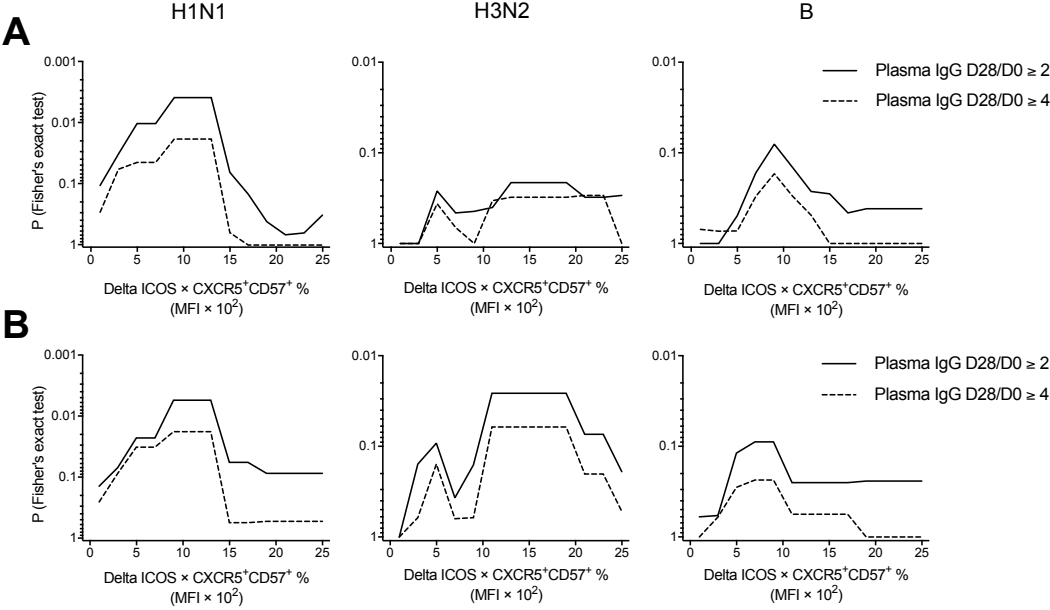

Figure S5

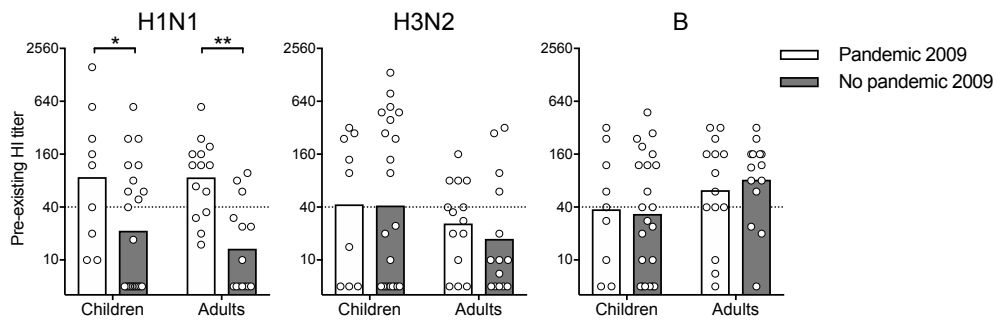

Figure S6

Supplement: jiz321_suppl_Supplementary_Figure [file jiz321_suppl_supplementary_figure.pdf]
