# Supplementary material for: Live-Attenuated Influenza Vaccine Induces Tonsillar Follicular T Helper Cell Responses That Correlate With Antibody Induction
Source: J Infect Dis. 2019 Jul 27;221(1):21–32. doi: 10.1093/infdis/jiz321 (PMC6910880; doi:10.1093/infdis/jiz321)
Supplement: jiz321_suppl_Supplementary_Figure_Legend [file jiz321_suppl_supplementary_figure_legend.docx]

Figure S1 The gating strategies of CD4^+^ T-cells in tonsillar mononuclear cells. An example is shown for a 4-year-old child (**A**) and a 40-year-old adult (**B**).

Figure S2 The CXCR5^+^CD57^+^ cells express the canonical T_FH_-cells markers. Representative expression of ICOS (**A**), PD1 (**B**), Bcl6 (**C**) and CD40L (**D**) in CXCR5^+^CD57^+^ (orange), CXCR5^+^CD57^-^ (blue), and CXCR5^-^CD57^-^ (red) T-cells in the same child (left) and adult (right) as in Figure 2C.

Figure S3 LAIV elicits long-term influenza-specific systemic antibodies in children. Total influenza specific systemic IgA (**A**) and IgM (**B**) were measured using plasma samples before (D0), 28 days (D28), 56 days (D56), 6 months (D180) and 12 months (D360) after vaccination. Antibodies were tested against antigens from A/California/07/2009-like (H1N1) virus (left panel) or A/Victoria/361/2011-like (H3N2) virus (central panel) or B/Massachusetts/2/2012 virus (right panel). The geometric mean values are shown as bars, and each symbol represents one subject. * P<0.05, ** P<0.01, *** P<0.001 (antibody concentrations were Ln transformed in statistical analyses. Sidak’s multiple comparisons between before and different days after vaccination were performed in two-way ANOVA).

Figure S4 Influenza-specific T_FH_-cell responses after LAIV correlate with antibody responses at day 56. The correlations between influenza-specific T_FH_-cell responses (Delta ICOS × CXCR5^+^CD57^+^ %) and systemic antibody responses (Plasma IgG D56/D0) against H1N1 (left panel), H3N2 (central panel) and B viruses (right panel). Data from all vaccinees (**A**), naïve individuals (D0 HI < 40 against H1N1 and H3N2, **B**), or pre-exposed individuals (D0 HI ≥ 40 against B virus, **B**) were included in the analyses. Systemic antibody fold inductions (Plasma IgG D56/D0) were Ln transformed in statistical analyses. Linear fitting curve was plotted as dotted line when nonparametric Spearman P < 0.10. Spearman r and P values are noted for each correlation.

Figure S5 Predictor capacity of LAIV induced T_FH_-cell responses. Different cutoffs of LAIV induced T_FH_-cell responses were tested for prediction of systemic antibody fold induction (Plasma IgG D28/D0 ≥ 2 (solid line), and ≥ 4 (dotted line)) at day 28 using Fisher’s exact test. Data from all vaccinees (**A**), naïve individuals (D0 HI < 40 against H1N1 and H3N2, **B**), or pre-exposed individuals (D0 HI ≥ 40 against B virus, **B**) were included in the Fisher’s exact test. Based on such tests, a total LAIV induced T_FH_-cell response of 900-1100 MFI is needed to predict a 2-fold induction of systemic IgG after vaccination in naïve individuals against influenza A viruses and in pre-exposed individuals against influenza B virus. T_FH_-cell responses and antibody fold induction were tested against split antigens from A/California/07/2009-like (H1N1) virus (left panel) or A/Victoria/361/2011-like (H3N2) virus (central panel) or B/Massachusetts/2/2012 virus (right panel).

Figure S6 The influence of pandemic vaccine 2009 on pre-existing HI titer. Pre-existing (D0) HI titers from individuals who received the pandemic vaccine in 2009 were plotted with the HI titers from those didn’t receive the pandemic vaccine in 2009. HI titers were tested against antigens from A/California/07/2009-like (H1N1) virus (left panel) or A/Victoria/361/2011-like (H3N2) virus (central panel) or B/Massachusetts/2/2012 virus (right panel). * P<0.05, ** P<0.01 (HI titers were Ln transformed in statistical analyses. Sidak’s multiple comparisons were performed in two-way ANOVA). The horizontal dotted lines indicate HI titer of 40.
