# Supplementary material for: Live-Attenuated Influenza Vaccine Induces Tonsillar Follicular T Helper Cell Responses That Correlate With Antibody Induction
Source: J Infect Dis. 2019 Jul 27;221(1):21–32. doi: 10.1093/infdis/jiz321 (PMC6910880; doi:10.1093/infdis/jiz321)
Supplement: jiz321_suppl_Supplementary_Table [file jiz321_suppl_supplementary_table.docx]

Supplementary table. The demographics of the subjects enrolled in the study

|  | Children | | | | | Adults | | | | |
| --- | --- | --- | --- | --- | --- | --- | --- | --- | --- | --- |
|  | Total | Control | Group 1 | Group 2 | Group 3 | Total | Control | Group 1 | Group 2 | Group 3 |
| No. of subjects  (No. of subjects with Tonsils) | 40  (25) | 6  (6) | 7  (5) | 15  (6) | 12  (8) | 37^2^  (23) | 6  (6) | 15  (6) | 8^1^  (5) | 8^1^  (6) |
| Gender, M/F | 23/17 | 3/3 | 4/3 | 10/5 | 6/6 | 9/28 | 0/6 | 3/12 | 3/5 | 3/5 |
| Median age^2^, years  (Range) | 4  (3-17) | 4.5  (3.5-8) | 11  (3-15) | 4  (3-13) | 4.25  (3-17) | 27  (18-51) | 23.5  (18-40) | 30  (19-51) | 24  (18-35) | 32.5  (18-42) |
| No. of subjects with LAIV,  one dose/ two doses | 7/27 | 0/0 | 4/3 | 1/14 | 2/10 | 31/0 | 0/0 | 15/0 | 8/0 | 8/0 |
| Median DT^3^ after LAIV, days  (Range) | 7.5  (2-22) | N/A^4^ | 4  (2-5) | 7  (6-9) | 14  (10-22) | 6  (2-21) | N/A^4^ | 4  (2-5) | 7  (6-9) | 14.5  (10-21) |
| Tonsillectomy indication,  Chronic tonsillitis/hypotrophy/both | 4/15/21 | 0/3/3 | 1/1/5 | 1/7/7 | 2/4/6 | 18/7/10^5^ | 2/0/3^5^ | 9/3/2^5^ | 5/1/2 | 2/3/3 |
| No. of subjects with previous vaccinations,  Pandemic 2009/ earlier seasonal vaccine | 9/4 | 0/0 | 1/0 | 3/2^6^ | 5/2 | 18/5 | 2/3 | 7/1 | 3/1 | 6/0 |

^1^ Three adults had only tonsils collected with no plasma or saliva samples, 1 in Group 2, 2 in Group 3.

^2^ Children younger than 5 were registered as 3, 3.5, 4 or 4.5 years old.

^3^ Days of tonsillectomy (DT) after receiving the live attenuated influenza vaccine.

^4^ Not applicable (N/A). Control subjects did not receive the live attenuated influenza vaccine before tonsillectomy.

^5^ Two subjects had indication for tonsillectomy unknown, one in Control, the other in Group 1.

^6^ Three subjects had unknown Pandemic 2009 vaccination history.
